# Asthma/COPD clinics increases adherence to management guidelines and associates with less morbidity and lower all-cause mortality – a prospective cohort study

**DOI:** 10.1038/s41533-026-00497-3

**Published:** 2026-03-06

**Authors:** Jenny Nilsson, Helena Backman, Johanna Karlsson Sundbaum, Viktor Strandkvist, Linnea Hedman, Caroline Stridsman

**Affiliations:** 1 Department of public health and clinical medicine, The OLIN and Sunderby Research Unit, Umeå University, Umeå, Sweden; 2 Department of health, medicine and rehabilitation, Luleå University of Technology, Luleå, Sweden

**Keywords:** Diseases, Health care, Medical research

## Abstract

In asthma, suboptimal disease control is common due to limited knowledge about self-management, undertreatment and infrequent follow-up visits. Most patients are treated in primary care where asthma/COPD clinics (ACC) are recommended in Sweden, but evidence of the effects is limited. The aim was to compare certified ACCs with clinics providing regular care in terms of adherence to asthma management guidelines, and the associations with asthma symptom control, healthcare consumption, and mortality in adults with asthma. In this cohort study, we extracted data from the Swedish National Airway Register, on 84230 adults with asthma, cared for at certified ACCs (n = 17 primary care centres) and regular care clinics (n = 650 primary care centres) in Sweden. Data were linked to other national registers in order to obtain data about pharmaceuticals, healthcare consumption, and mortality. The index date was the years 2015–2017, and the study ended in 2022. A binary logistic regression was used to assess morbidity and mortality associations at the study’s end. A higher proportion of patients at certified ACCs received interventions such as patient education, written asthma action plan, smoking cessation, Asthma Control Test, spirometry, and inhaled corticosteroids than patients at regular care clinics. Certified ACCs were associated with a lower probability of uncontrolled asthma (OR 0.76, 95% CI 0.67–0.87), need of specialist/emergency care (OR 0.69, 95% CI 0.51–0.92) and death (OR 0.69, 95% CI 0.55–0.86). In conclusion, adherence to asthma management guidelines was higher in certified ACCs which were associated with a more well-controlled asthma, less secondary healthcare visits and lower all-cause mortality, but not with frequent exacerbations. Our findings highlight the importance of ACCs in providing evidence-based care in accordance with asthma management guidelines.

## Introduction

Asthma is one of the most common non-communicable public health diseases globally, with a prevalence of approximately 10% in high-income countries. Asthma is characterised by airway inflammation and variable bronchial obstruction with symptoms including dyspnoea, cough, chest tightness and wheezing^1,2^. Treatment goals include absence of daily asthma symptoms, prevention of exacerbations, and maintenance of normal lung function^2,3^, however, a large proportion of patients with asthma do not reach the treatment goals and therefore have an uncontrolled disease^4–6^. Uncontrolled asthma is associated with an increased risk of exacerbation and healthcare utilisation, decreased quality of life^5,7^, mortality, and societal and individual costs^5,8^. According to management guidelines, asthma should be regularly assessed with regard to treatment and asthma control. Patients should also be offered patient education including self-management, smoking cessation support and written asthma action plans^2,3,9^.

Most adult patients with asthma are diagnosed and treated in primary care^2–4,9–11^. To ensure high-quality and structured asthma care, the recommendation for Swedish primary care is to adhere to asthma management guidelines and to organise asthma/chronic obstructive pulmonary disease (COPD) clinics (ACC)^3,9^. However, regular asthma follow-up visits are rare among adults^5,10,12^, most prescription renewals occur via telephone, at which point asthma symptoms are not assessed^12^. Furthermore, knowledge about asthma and asthma control is lacking among patients, and both undertreatment and low adherence are common^1,6,9^. Barriers to treatment adherence, as perceived by patients with asthma, include deficient physician–patient communication, lack of knowledge about asthma, and insufficient involvement in treatment decisions^13^.

Self-management and shared decision-making can improve asthma outcomes^2,5,14^. Accordingly, the key responsibilities in a Swedish certified ACC are to conduct regular follow-up visits involving assessment of symptoms and adjustment of non-pharmacological and pharmacological treatment, to strengthen self-management, and to establish written asthma action plans in cooperation with the patient^15,16^. For COPD, ACCs have been shown to have positive effects by lowering the frequency of exacerbations and overall treatment costs^17^. By contrast, a systematic review found minor effects on healthcare use, quality of life, and medication use among patients with asthma^18^, but evidence in the review was inconclusive due to the few available studies. Further research about ACCs is important, especially regarding their potential health benefits for patients with asthma^18,19^. Therefore, the aim of this study was to compare certified asthma/COPD clinics with clinics providing regular care in terms of adherence to asthma management guidelines, and the associations with asthma symptom control, healthcare consumption, and mortality in adults with asthma.

## Material & methods

### Study design

This is a register-based cohort study with a prospective design.

### Study setting and population

The Swedish National Airway Register (SNAR) is a quality register of patients with asthma and/or COPD registered in primary and secondary care. The register was initiated in 2013, and as of 2017, all regions in Sweden were affiliated to the register, covering over 900 primary care centres^20^. The current study was based on primary care data (generally directly transferred from the medical records to SNAR) between 2015 and 2022. After the exclusion of patients with concomitant COPD (n = 5970, International Statistical Classification of Diseases and Related Health Problems 10^th^ revision (ICD-10) J.44), a total of 84230 patients aged 18 years or older with physician diagnosis of asthma (ICD-10 J.45) were identified in 2015–2017 (index date). These patients were followed until 31 December 2022 (end of study period).

In Sweden, patients have the legal right to choose their healthcare provider, as stipulated in the Patient Act (2014:821)^21^. Primary care centres are typically dominated by general practitioner consultations, while ACCs are integrated as part of the centres and have adopted the clinic structure as recommended^15,16^. At index date (2015–2017) 667 primary care centres were identified in SNAR. Of those, 17 centres had certified ACCs for the entirety of the period, all located in different areas in Region Skåne, the southernmost part of Sweden. Patients in ACCs were compared to patients in the other primary care centres (n = 650), spread across Sweden, further on referred to as regular care clinics. In contrast to other regions in Sweden, Region Skåne has actively worked on annual certifications of asthma/COPD clinics through questionnaires since 2015. To be certified, several criteria must be met [Supplementary Table S1]. For one, clinics should be grounded in team integration with a person-centred approach, led by a nurse specialised in asthma/COPD with at least 15 university credits at the master’s level and with allocated time. In addition to the nurse, a general practitioner with operational responsibility, a physiotherapist, a dietician, an occupational therapist and a psychologist should be included in the team. Moreover, patients in ACCs have a general practitioner at the primary care centre responsible for diagnosis, treatment and follow-up as in regular care clinics. Patients should be provided with both pharmacological and non-pharmacological interventions in accordance with asthma management guidelines. Finally, provided care should be documented in SNAR^22^, as the national quality register in Sweden serves as a measure of quality in asthma management^20^.

### Data collection

**Fig. 1:**
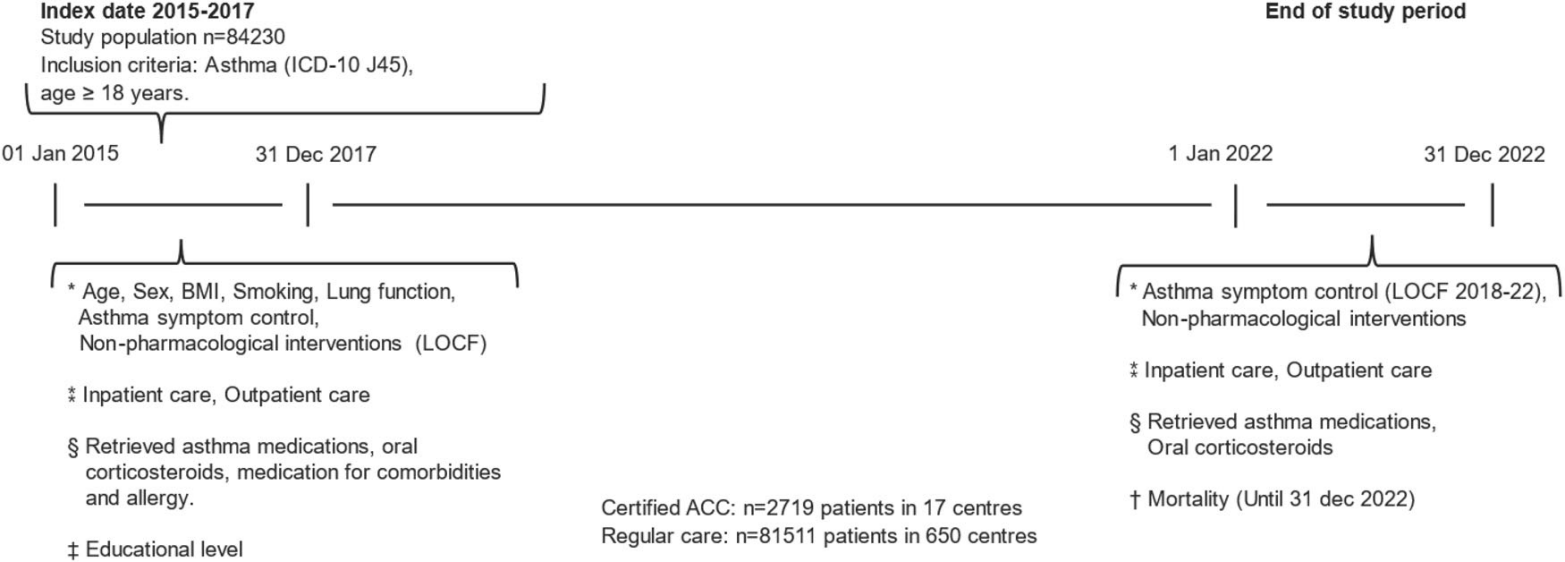
Timeline, study design and data collection. Abbreviations: ICD-10, International Statistical Classification of Diseases and Related Health Problems – Tenth revision; BMI, Body Mass Index. LOCF, Last Observation Carried Forward. Notes: * Swedish National Airway Registry (SNAR), ⁑ National Patient Registry (NPR), § National Prescribed Drug Registry (NPDR), † National Cause of Death Registry (NCDR), ‡ Longitudinal Integrated Database for Health Insurance and Labor Market Studies (LISA).

Data on age, sex, smoking habits, body mass index (BMI), lung function, Asthma Control Test (ACT), spirometry, patient education, written asthma action plans, and smoking cession support were retrieved from SNAR at index date (baseline) and study end. If patients had multiple registrations in the identified clinic affiliation during the index period, data from the last registration was used. If data on individual variables was missing at inclusion, the last observation carried forward method (LOCF) was used to obtain as complete data as possible. Data on educational level were retrieved from the Longitudinal Integrated Database for Health Insurance and Labor Market Studies (LISA)^23^; pharmacological treatment data were retrieved from the National Prescribed Drug Register (NPDR); and healthcare consumption data were retrieved from the National Patient Register (NPR) at baseline and study end. Data on mortality were retrieved from the National Cause of Death Register (NCDR) until the study’s end^24^ [Fig. 1].

### Definitions

*Educational level* was categorised as primary (9 years of school), secondary (12 years of school), and tertiary (>12 years of school) education. *Comorbidities and allergy were* identified using dispensed medication, based on Anatomical Therapeutic Chemical Codes (ATC-codes); thus, the dispensed medication was a proxy for cardiovascular disease, depression/anxiety, diabetes, and allergy [Supplementary Table S2]. *BMI* (kg/m^2^) was categorised into normal weight (BMI ≤ 24.9), overweight (BMI 25–29.9), and obesity (BMI ≥ 30). The proportion of underweight patients was small, so these patients were analysed together with patients of normal weight. *Smoking habits* were categorised as current, former and never smoker. *Lung function* was measured through Forced Expiratory Volume in 1 second (FEV_1_) expressed as percent of predicted (pp), and was categorised into FEV_1_ < 80 pp and FEV_1_ ≥ 80 pp post-bronchodilation: if post-bronchodilation values were missing, pre-bronchodilation values were used. The Swedish Hedenström reference values for lung function tests were used^25^. *Asthma symptom control* was assessed by ACT, a validated questionnaire with scores ranging from 5–25. Well-controlled asthma was defined as ACT ≥ 20, and uncontrolled asthma as ACT ≤ 19^26^. *Frequent exacerbations* were defined as oral corticosteroids (OCS) being dispensed on ≥2 occasions. *Healthcare consumption* was defined as inpatient (hospitalisation) and outpatient (specialist clinics and emergency visits) care due to asthma or status asthmaticus (ICD-10 J45, J46). *Patient education, asthma action plan and smoking cessation support* were defined as affirmative responses to the following questions in SNAR: “Has the patient received structured patient education the past 5 years (including disease knowledge, self-management, risk factors, medication and inhalation technique)?”, “Has the patient been provided with an asthma action plan?” and “Has the patient been offered smoking cessation support?”. *Pharmacological asthma treatment* was defined by ATC-codes [Supplementary Table S1]. Inhaled corticosteroids (ICS) and long-acting β _2_-agonist (LABA) were categorised as separate inhalers and fixed combination inhalers. Short acting β _2_-agonist (SABA) was categorised as monotherapy. *Mortality* was defined as death from any cause occurring during the study period.

### Statistical methods

IBM Statistical Package for Social Sciences (SPSS), version 29.0.1.0 was used for the analysis. Descriptive data of baseline characteristics, pharmacological treatment and non-pharmacological interventions were presented as frequencies (%) for categorical data, and as mean values and standard deviations (SD) for continuous data. Comparisons between groups were analysed by using a chi-square test or an independent samples t-test, and a p-value < 0.05 was considered statistically significant. Univariate and multivariate logistic regression models were used to analyse the associations between attendance at certified ACCs and future asthma symptom control, exacerbation, healthcare consumption and all-cause mortality at study end, by calculating odds ratios (OR) and 95% confidence intervals (CI). All models were adjusted for potential confounders of age, sex, cardiovascular disease, BMI, and smoking habits at baseline.

### Ethical approval

In accordance with Swedish regulations governing national quality registers the opt-out model was used and informed consent was not required. Patients are informed about the register and can withdraw their data at any time. The study was conducted according to the Declaration of Helsinki and approved by the Swedish Ethical Review Authority (dnr 2019-04915, dnr 2020-00508).

## Results

### Characteristics

Of n = 84230 included patients with asthma in primary care, n = 2719 (3.2%) were cared for at certified ACCs and n = 81511 (96.8%) at regular care clinics. Patient characteristics at baseline are shown in Table 1. Mean age was 49.4 vs. 50.8 years, and the proportion of women 60.2% vs. 62.5% at certified ACCs vs. regular care clinics, respectively. Patients at certified ACCs had slightly less medication for comorbidities such as cardiovascular disease, depression/anxiety, and allergies, and a larger proportion were of normal weight and were former smokers than patients of regular care clinics. A lower proportion of patients at certified ACCs had FEV_1_ < 80 pp and uncontrolled asthma compared to patients at regular care clinics. Frequent exacerbations occurred in almost one–fifth of all patients at baseline, with no differences seen between the groups. The proportion of healthcare consumption was low overall. At baseline, less than 1% of all patients had been hospitalised, and 9.4% of patients had visits to specialist or emergency care. Both hospitalisation and specialist/emergency care were more common among patients at regular care clinics than those at certified ACCs.

**Table 1:** Patient characteristics at baseline, among all and among patients in certified ACC and regular care clinics, year 2015–2017.

	All (n = 84230)	Certified ACC (n = 2719, 3.2%)	Regular care clinics (n = 81511, 96.8%)	p-value
**Age**, mean (SD, min-max)	50.8 (18.6, 18–106)	49.4 (18.6, 18–93)	50.8 (18.6, 18–106)	**<0.001**
**Sex**				
Female	52602 (62.5)	1637 (60.2)	50965 (62.5)	**0.014**
**Educational level**				
Primary	12475 (15.6)	400 (15.2)	12075 (15.6)	0.891
Secondary	34263 (42.8)	1127 (42.9)	33136 (42.8)	
Tertiary	33344 (41.6)	1097 (41.8)	32247 (41.6)	
Missing	4148 (4.9)	95 (3.5)	4053 (5.0)	
**Medication for comorbidities**				
Depression/Anxiety	20139 (23.9)	554 (20.4)	19585 (24.0)	**<0.001**
Diabetes	6721 (8.0)	190 (7.0)	6531 (8.0)	0.052
Cardiovascular disease	29229 (34.7)	799 (29.4)	28430 (34.9)	**<0.001**
**Allergy**	37423 (44.4)	1123 (41.3)	36300 (44.5)	**<0.001**
**Body Mass Index** (BMI)				
BMI, mean (SD)	27.8 (5.7)	27.6 (5.6)	27.8 (5.7)	**0.030**
Normal BMI < 25	18954 (34.2)	902 (36.5)	18052 (34.1)	**0.020**
Overweight BMI 25-29.9	19833 (35.8)	877 (35.5)	18956 (35.8)	
Obesity, BMI ≥ 30	16685 (30.1)	692 (28.0)	15993 (30.2)	
Missing	28758 (34.1)	248 (9.1)	28510 (35.0)	
**Smoking Habits**				
Current smoker	6497 (10.9)	271 (11.5)	6226 (10.8)	**<0.001**
Former smoker	11963 (20.0)	624 (26.6)	11339 (19.7)	
Never smoker	41304 (69.1)	1452 (61.9)	39852 (69.4)	
Missing	24466 (29.0)	372 (13.7)	24094 (29.6)	
**Lung function** (after bronchodilation)				
FEV_1_% pred, mean (SD)	83.8 (16.1)	84.9 (15.9)	83.7 (16.1)	**<0.001**
FEV_1_ < 80%	13140 (36.6)	803 (34.4)	12337 (36.8)	**0.025**
Missing	48334 (57.4)	388 (14.3)	47946 (58.8)	
**Asthma Control Test** (ACT)				
ACT score, mean (SD)	19.8 (4.4)	20.1 (4.4)	19.8 (4.4)	**0.022**
ACT ≤ 19	10530 (37.7)	662 (34.6)	9868 (37.9)	**0.004**
Missing	56273 (66.8)	804 (29.6)	55469 (68.1)	
**Frequent exacerbations** (OCS courses ≥ 2)	16701 (19.8)	546 (20.1)	16155 (19.8)	0.737
**Healthcare consumption**				
Hospitalisation	669 (0.8)	9 (0.3)	660 (0.8)	**0.006**
Specialist and emergency care	7955 (9.4)	148 (5.4)	7807 (9.6)	**<0.001**

Numbers are presented as n (%) unless specified otherwise; Lung function values, after bronchodilation; Statistical tests used, except descriptives, were t-test and chi-square test; P-values comparing certified ACC with regular care clinics, significant p-values (p < 0.05) in bold font; Missing data: Described as n (%).

*ACC*, asthma/COPD clinic; *SD*, standard deviation; *FEV*_*1*_*% pred*, Forced Expiratory Volume in 1 s as percent of predicted value; *ACT*, asthma control test; *OCS*, oral corticosteroids.

### Certified ACCs’ adherence to non-pharmacological and pharmacological guidelines at baseline

The certified ACCs conducted and offered all non-pharmacological interventions to a greater extent than regular care clinics. The proportion of their patients receiving structured patient education was three times higher, and twice as many patients had answered the ACT and performed spirometry at certified ACCs than at regular care. Written asthma action plans were established, and smoking cessation support was offered to current smokers to a larger proportion of patients of certified ACCs than those of regular care clinics [Table 2].

**Table 2:** Non-pharmacological and pharmacological treatment at baseline, year 2015–2017.

	All (n = 84230)	Certified ACC (n = 2719, 3.2%)	Regular care clinics (n = 81511, 96.8%)	p-value
**Non-pharmacological intervention**				
Asthma Control Test	27957 (33.2)	1915 (70.4)	26042 (31.9)	**<0.001**
Spirometry	25023 (29.7)	1956 (71.9)	23067 (28.3)	**<0.001**
Structured patient education	18928 (22.5)	1712 (63.0)	17216 (21.1)	**<0.001**
Written asthma action plan	3027 (3.6)	396 (14.6)	2631 (3.2)	**<0.001**
Smoking Cessation Support	1541 (23.7)	95 (35.1)	1446 (23.2)	**<0.001**
**Pharmacological treatment**				
SABA as monotherapy	6562 (7.8)	102 (3.8)	6460 (7.9)	**<0.001**
Any ICS*	73630 (87.4)	2534 (93.2)	71096 (87.2)	**<0.001**
Any ICS/LABA^¤^	43622 (51.8)	1639 (60.3)	41983 (51.5)	**<0.001**
*ICS/LABA fixed combination*	37259 (44.2)	1480 (54.4)	35779 (43.9)	**<0.001**
*ICS/LABA separate inhalers*	6363 (7.6)	159 (5.8)	6204 (7.6)	**<0.001**
LTRA	11538 (13.7)	398 (14.6)	11140 (13.7)	0.147

Numbers are presented as n (%). The p-value comparing certified ACCs with regular care clinics, significant p-values (p < 0.05) in bold font.* ICS, ICS/LABA or ICS/LABA/LAMA, with or without SABA; ^¤^ Separate inhalers or in fixed combination; Statistical analysis, descriptives and chi-square test.

*ACC*, asthma/COPD clinic; *SABA*, Short-acting β-agonists; *ICS*, inhaled corticosteroids; *ICS/LABA*, Inhaled corticosteroids in combination with long-acting β-agonists; *LTRA*, Leukotriene receptor antagonists.

SABA as the only inhaled medication, was approximately twice as commonly dispensed to patients at regular care clinics than those of certified ACCs. Dispensation of any ICS and ICS/LABA in fixed combination was more commonly dispensed to patients of certified ACCs, while ICS/LABA in separate inhalers were more commonly dispensed to patients at regular care clinics. Leukotriene receptor antagonists were dispensed to patients similarly across groups [Table 2].

### Certified ACCs and associations with asthma symptom control, exacerbation, healthcare consumption and mortality

At the study end in 2022, mean ACT scores increased among those who had answered the ACT. At certified ACCs, a higher proportion of patients had well-controlled asthma compared to patients at regular care clinics. There were no differences between certified ACCs and regular care clinics in terms of proportions of patients with frequent exacerbations and hospitalisation. In both groups, hospitalisation was still uncommon. At regular care clinics, a higher proportion of patients visited a specialist clinic or emergency care compared to patients of certified ACCs. The proportion of all-cause mortality was higher for regular care clinics than for ACCs [Table 3].

**Table 3:** Asthma symptom control, exacerbation, healthcare consumption and mortality at the study end, year 2022.

	All (n = 84230)	Certified ACC (n = 2719, 3.2%)	Regular care clinics (n = 81511, 96.8%)	p-value
**Asthma Control Test**				
ACT mean points (SD)	20.1 (4.5)	20.8 (4.4)	20.1 (4.5)	**<0.001**
ACT ≤ 19	8972 (35.3)	324 (28.4)	8648 (35.6)	**<0.001**
Missing	58820 (69.8)	1580 (58.1)	57240 (70.2)	
**Frequent exacerbations**				
OCS courses ≥ 2	5716 (6.8)	195 (7.2)	5521 (6.8)	0.416
**Healthcare consumption**				
Hospitalisation	87 (0.1)	1 (0.0)	86 (0.1)	0.272
Specialist and emergency care	2049 (2.4)	47 (1.7)	2002 (2.5)	**0.015**
**Deaths**	4710 (5.6)	92 (3.4)	4618 (5.7)	**<0.001**

Numbers are presented as n (%) unless otherwise specified. The p-value comparing certified ACCs with regular care clinics, significant p-values (p < 0.05) in bold font. Statistical analysis, descriptives and chi-square test.

*ACC*, asthma/COPD clinic; *ACT*, asthma control test; *OCS*, Oral Corticosteroids; *SD*, standard deviation.

**Fig. 2:**
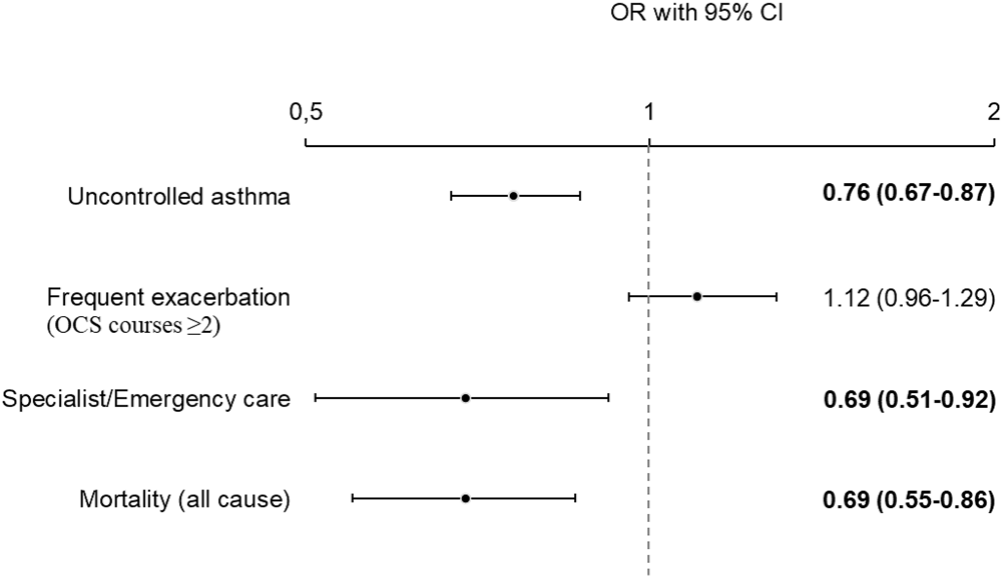
Certified asthma/COPD clinics and the associations with uncontrolled asthma= ACT ≤ 19 (model 1), frequent exacerbations= OCS ≥ 2 (model 2), healthcare consumption (model 3) and mortality (model 4) by four logistic regression models. All models were adjusted for age, sex, medication for cardiovascular disease, Body Mass Index and smoking habits. Results presented as odds ratios (OR) with 95% confidence intervals (CI), significant p-values (p < 0.05) in bold font.

After adjustment for sex, age, medication for cardiovascular disease, BMI, and smoking habits, receiving care at a certified ACC was associated with a lower probability of uncontrolled asthma (OR 0.76, 95% CI 0.67–0.87), having specialist or emergency visits (OR 0.69, 95% CI 0.51–0.92), and death (OR 0.69, 95% CI 0.55–0.86) in 2022. No association was found between certified ACCs and frequent exacerbations (OR 1.12, 95% CI 0.96–1.29) [Fig. 2, model 1-4]. Crude and adjusted ORs of associations between certified ACCs and the outcomes of asthma symptom control, frequent exacerbations, specialist or emergency visits, and mortality are shown in Table S3.

## Discussion

A total of 84230 patients were included in this nationwide register-based study. The findings showed that certified ACCs adhered to asthma management guidelines to a greater extent, with regard to both pharmacological treatment and non-pharmacological interventions, compared to clinics providing regular care. Certified ACCs were also associated with better health outcomes such as well-controlled asthma, less need for specialist and emergency care visits and a lower probability of all-cause mortality. This study contributes knowledge on and highlights the importance of prioritising asthma care and of organising ACCs to enable high-quality, evidence-based, and equitable primary care, in line with other studies^11,17,27–29^. Moreover, care at ACCs is both cost effective^17,29^ and aligns with the person-centred and integrated care vision which politicians in Sweden decided is a goal for future healthcare^30^.

### Adherence to management guidelines

Earlier research has demonstrated the advantages of providers working in a team, of staff having asthma specific education, and of implementing treatment in line with current guidelines^27,31^. For example, primary care organised with nurses at the centre of management has been indicated to adhere better to guidelines than regular care clinics^31^, as consistent with our study. Patients of ACCs have access to a specialist nurse, improving continuity in care with a person-centred, transdiagnostic and holistic approach^32,33^. Moreover, current asthma management guidelines emphasize multimorbidity management^2^, both mitigating the risk of overlooking multimorbidity. Additionally, attendance at ACCs includes a general practitioner contact^15^. Moreover, regular care clinics had lower adherence, which may be due to lack of knowledge, local routines, and time pressure^34^. At the same time, the Continuity of Care Index in Swedish primary care has decreased over time^35^. Nevertheless, even though certified ACCs in this study adhered to the non-pharmacological interventions to a greater extent than did regular care clinics, the target levels in the Swedish guidelines have not yet been fully attained. Spirometry at diagnosis, ACT, and smoking cessation support should be performed with 95% of all patients, and patient education with 80%^9^. Our results indicate that continued efforts are required to reach these goals.

At baseline, there were statistical differences in some characteristics, likely with a minor clinical relevance concerning asthma symptom control, lung function and comorbidities. However, spirometry was carried out with only approximately 30% of patients at regular care clinics, while the numbers was more than twice as high for patients at certified ACCs. The same trend was seen concerning ACT, wherein a substantially higher proportion of patients at ACCs had their asthma symptom control evaluated. These findings are consistent with other studies showing that the use of spirometry and tools to assess asthma control and symptoms were low^28,36,37^. The findings regarding asthma care at regular care clinics are worrying, given that these interventions are important not only for diagnosis but also for regular evaluation of treatment and risk assessment for exacerbations^2,3^. Furthermore, more than one-third of current smokers were offered smoking cessation support at certified ACCs, compared to less than one-fourth at regular care clinics. Smoking cessation support is an intervention which should be more widely accessible to smokers with asthma^2,8,38^, since smoking is known to be related to hospitalisation, poor symptom control, and reduced efficacy of ICS^2,12,38^.

In our study, three times as many patients received patient education at certified ACCs compared to patients at regular care clinics. This is consistent with previous studies, in which patients of certified ACCs received a more extensive education and thereby obtained more knowledge on asthma and self-management^11,29^. Patient education is known to be associated with better adherence to ICS treatment^6^, better asthma control^11,39^, fewer emergency care visits, and fewer hospitalisations^29,40^. With more time allocated for the nurse^11^, patients’ knowledge as well as asthma control increases. However, the time allocated for nurses in the current study is unknown. Additionally, the use of written asthma action plans was more common at certified ACCs, in line with earlier findings^29^. In our study, only 15% of patients at certified ACCs received an asthma action plan, although this was higher than the 3% among patients at regular care clinics. Low proportions for asthma action plans were also reported in another Swedish study (6%)^20^, and a US study (3%)^41^. Thus, written asthma action plans need to be used to a higher extent, both in Sweden and internationally, since this intervention strengthens self-management and subsequently reduces unscheduled healthcare visits and hospital admissions as well as improves asthma control and quality of life^3,12,14^.

Other studies have shown that poor adherence to pharmacological treatment guidelines is common in primary care^42,43^. In our study, ICS treatment was more prevalent for patients of certified ACCs, which also prescribed fixed combinations of ICS/LABA (instead of two separate inhalers) to a larger extent than regular care clinics. Furthermore, a higher proportion of patients of regular care clinics used SABA as monotherapy. Our findings suggest that certified ACCs possess better medication knowledge and prescribe more in line with guideline recommendations than regular care clinics. A simplified treatment, such as a fixed combination inhaler, enhances patients’ medication adherence and inhalation technique, which in turn decreases the risk of adverse events^1,6,44–46^. In addition, medication adherence has previously been found to be better in patients of ACCs than patients of regular care clinics^29^.

### Associations with morbidity and all-cause mortality

In contrast to a previous study finding no differences in asthma control related to the organisation of care^11^, we found that receiving care at a certified ACC was associated with future well-controlled asthma. One explanation could be the use of different definitions of controlled asthma, as the previous study also included the concept of exacerbations. Importantly, though, uncontrolled asthma is common^4–6,47^, and in our study a high proportion of patients in both groups had uncontrolled asthma (28–36%). At the same time, we also found that patients of certified ACCs had less need for specialist and emergency care, in line with other studies^29,40^. It has been postulated that healthcare professionals in certified ACCs are more knowledgeable about asthma and therefore a decreased need to refer patients to a higher level of care^17^. Other than that, the difference could be due to patients having more knowledge about asthma and self-management^11,14^.

With regard to COPD exacerbations, ACC attendance has been shown to be a protective factor^17^, but not in our study which concerned asthma, where no associations with ACCs were found. A possible explanation for this might be that primary care centres with certified ACCs might be better at identifying and treating exacerbations than the other centres. Frequent asthma exacerbations occurred in 20% of patients at baseline but were halved by 2022. This is probably an effect of the SARS-CoV-2 (COVID-19) pandemic^48^, and it may have influenced the result, as virus exposure is a large explanatory factor for asthma exacerbations^1^, and pandemic restrictions reduced exposure^49,50^. The pandemic may also have affected hospitalisations due to asthma being uncommon, but due to the low number of patients, we could not conduct correlation analyses. However, pre-pandemic studies have demonstrated associations between ACCs and less need of hospitalisation for COPD^17^. For asthma, this finding has not been substantiated^29^, which may be due to decreased occurrence of asthma related hospitalisations over the years, even pre-pandemic^24^.

As asthma treatment has developed, asthma mortality rates have declined^2,5,8^, therefore, our study only included all-cause mortality. During the study period 5.6% of the patients died, and certified ACCs were associated with lower probability of death. Similar proportions (3.5-5.3%) were seen in another study, albeit with higher rates in patients with severe asthma^51^. Yet, another study in Norway and Sweden found that 5.5% of all deaths could be linked to current asthma with an increased risk of death by 71% compared to those who do not have asthma^52^. Our study is the first to investigate the association between certified ACCs and all-cause mortality in patients with asthma, thereby adding new knowledge concerning healthcare provided in certified ACCs. Nevertheless, further studies are needed to deepen the understanding of causality in this context.

### Strengths and limitations

This observational study, with its prospective design and large study population, reflects clinical reality in Swedish asthma care. The Swedish health registries used in this study have a high coverage rate, and the Swedish individual social security number enables data linkage. During all studied years, most of the data was directly transferred from medical records to SNAR, which minimises the risk of reporting bias. Due to the certification process in Region Skåne, we were able to ensure that the clinics included in the certified ACC group met the requirements of an ACC as outlined in Swedish asthma management guidelines^9^. Furthermore, the validated ACT was used to assess asthma symptom control^26^.

Our study also has limitations. To start, the two compared groups were substantially unequal in size, which could affect the precision and the risk of bias. Nevertheless, the smaller group (ACC patients) demonstrated significant associations with beneficial health outcomes, indicating a robust effect. Moreover, the representativeness is important. Earlier studies found that the distribution of ACCs across Sweden and their criteria fulfilment vary^11,17,28^. Since the initiation of SNAR, there was variability across regions in the proportion of affiliated clinics and registration rates; thus, all the reporting clinics might have had a higher adherence to asthma guidelines. In addition, in our study, a geographically limited group was compared with a group clinics spread across the country, although with similar socioeconomic status when using educational level as a proxy. Finally, primary care centres organised as ACCs might also have been included in the group of regular care clinics, since a national system for ACC identification is lacking. All these points lead to a possible selection bias. Another important point is that patients may have been treated in both types of clinics during the study period, which could not be verified if the patient changed to a healthcare provider not affiliated to SNAR.

For limitations related to definitions and outcomes, pharmacological treatments were defined by dispensed prescriptions, which might differ from actual medication use. For the latter, information about inhalation technique was also unknown. The use of medication dispensation based on ATC-codes as a proxy for comorbidities and exacerbations could also have led to under- or overestimations, due to comprehensiveness. Although similar definitions of comorbidities have been used, and the prevalences was aligned with other studies^6,53,54^. Specifically, the definition of exacerbations as dispensed OCS has been used previously^6^ and is validated for COPD^55^, yet OCS might have been dispensed for other indications than asthma. In addition, the COVID-19 pandemic may have affected the outcomes of this study; the frequencies of exacerbation and hospitalisation were low and may have been insufficient to be able to detect differences and associations. Moreover, ACCs were paused during the pandemic, and few patients had follow-up visits in primary care overall. Finally, the trend that certified ACCs adhered to guidelines to a greater extent than regular care clinics remained in 2022. Due to the high proportion of missing data in both groups, during the pandemic, the results could not be interpreted more closely.

Despite limitations, the coverage of SNAR has developed during the study period^20^; even though not all primary care centres are affiliated with SNAR, all regions in Sweden were represented, as well as both rural and urban areas of the Region Skåne. This, together with the few exclusion criteria and the large sample size, minimises the risk of bias, since the study includes a large heterogenous population. The findings in our study reflect patients with a physician diagnosis of asthma in a primary care setting in Sweden, and may be generalisable also to other countries with a similar organisation of primary care.

## Conclusion

In conclusion, primary care centres organised as certified ACCs maintain greater adherence to asthma guidelines than regular care clinics and are associated with a more well-controlled asthma, less need of emergency and specialist care, and a lower all-cause mortality, but not with frequent exacerbations. Our findings highlight the importance of ACCs in ensuring evidence-based care in accordance with asthma management guidelines. However, national target levels have not been achieved, and so continued efforts and a structure for implementation are needed.

## Supplementary information

Supplementary Tables

## Data Availability

Access to the data may be granted upon reasonable request, contingent upon approval by the Swedish Ethical Review Authority. Data is held by Region Norrbotten, in Luleå, Sweden.
