# Supplementary material for: Asthma/COPD clinics increases adherence to management guidelines and associates with less morbidity and lower all-cause mortality – a prospective cohort study
Source: NPJ Prim Care Respir Med. 2026 Mar 6;36:17. doi: 10.1038/s41533-026-00497-3 (PMC12992697; doi:10.1038/s41533-026-00497-3)
Supplement: Supplementary file 1 — Supplementary Tables [file 41533_2026_497_MOESM1_ESM.docx]

**SUPPLEMENTARY TABLES**

TABLE S1. Criteria for the annual certification process of asthma/COPD-clinics, Region Skåne.

|  | **Asthma/COPD and allergy nurse** | **General practitioner** |
| --- | --- | --- |
| **Competence** | At least 15 university credits at the master’s level about asthma/COPD and allergy | Specialist in Family medicine |
|  | Spirometry education/licence | |
| **Continuing education** | 1-2 days/year | 2 days/year |
| **Allocated time** | A minimum of 2 hours/week/1000 listed patients | 1-2 hours/week/1000 listed patients |
| **Interprofessional cooperation** | Physiotherapist, dietician, occupational therapist, curator/psychologist, certified smoking cessation counsellor | |
| **Equipment** | Spirometer, pulse oximeter, peak-flow meters, FEV_1_/FEV_6_ meter, oxygen, equipment for inhaled medication and allergy testing, information/education material and inhalers for demonstration | |
| **Working methods** | Person-centred approach  Structured investigation, assessment, treatment interventions, conduct follow-up visits  Measure lung function, offer smoking cessation support, establish written treatment plans, provide patient education, and smoking cessation support | |
| **Quality register** | Continuous registration of visits in the Swedish National Airway Registry. | |

Notes: The Region Skåne certification criteria are based on Swedish asthma/COPD management guidelines ^1, 2, 3^, and have continuously been revised.

1. National Board of Health and Welfare. *Nationella riktlinjer för vård vid astma och KOL: stöd för styrning och ledning.* National Board of Health and Welfare, Stockholm (2015).

2. Kull, I. et al. Efficient care in asthma/COPD primary health care clinics. *Läkartidningen*. **105,** 2937–2940 (2008).

3. Kull I, Ställberg B. New criteria for asthma/COPD clinics in primary care. *Läkartidningen*. **115,** 1–3 (2018).

TABLE S2. ATC-codes used to define comorbidity, allergy and pharmacological asthma treatment at baseline (January 2015-December 2017).

|  | **ATC-code** |
| --- | --- |
| **Medication for comorbidity** |  |
| Cardiovascular disease | C01-03, C08-09 |
| Depression/Anxiety | N06 |
| Diabetes | A10A, A10B |
| **Allergy** | R06 |
| **Inhaled asthma medication** |  |
| Inhaled Corticosteroid (ICS) | R03BA |
| Long acting β-agonist combination (LABA) | R03AC12-13, 18-19 |
| Long-acting muscarinic antagonist (LAMA) | R03BB04-07 |
| ICS/LABA * | R03AK06-08, 10-11, 14 |
| ICS/LABA/ LAMA * | R03AL08-09, 11-12 |
| Short-acting β-agonist (SABA) | R03AC02-03 |
| **Other asthma medication** |  |
| Leukotriene receptor antagonist (LTRA) | R03DC |
| Oral corticosteroid (OCS) | H02AB |

Notes: ATC-codes used to define comorbidity, allergy, asthma treatment and exacerbations; * Fixed combinations.

TABLE S3. Crude and adjusted odds ratios for variables in the logistic regression models of associations with the outcomes; Asthma Control, exacerbation, healthcare consumption, and all-cause mortality.

|  | **Uncontrolled asthma** | | **Frequent exacerbations** | | **Specialist and emergency care** | | **Mortality** | |
| --- | --- | --- | --- | --- | --- | --- | --- | --- |
|  | *Crude values* | *Adjusted values* | *Crude values* | *Adjusted values* | *Crude values* | *Adjusted values* | *Crude values* | *Adjusted values* |
| **Type of clinic** |  |  |  |  |  |  |  |  |
| Certified ACC | **0.72 (0.63–0.82)** | **0.76 (0.67–0.87)** | 1.06 (0.92–1.23) | 1.12 (0.96–1.29) | **0.70 (0.52**–**0.94)** | **0.69 (0.51**–**0.92)** | **0.58 (0.47**–**0.72)** | **0.69 (0.55**–**0.86)** |
| **Demographic characteristics** |  |  |  |  |  |  |  |  |
| Female sex | **1.46 (1.39**–**1.55)** | **1.46 (1.38**–**1.54)** | **1.42 (1.34**–**1.51)** | **1.38 (1.30**–**1.47)** | **1.21 (1.10**–**1.33)** | **1.23 (1.12**–**1.35)** | 0.99 (0.93–1.05) | **0.68 (0.63**–**0.73)** |
| Older age | 1.00 (1.00–1.00) | **1.00 (0.99**–**1.00)** | **1.02 (1.02**–**1.02)** | **1.01 (1.01**–**1.01)** | **1.0 (0.99**–**1.00)** | **0.99 (0.99**–**1.00)** | **1.13 (1.12**–**1.13)** | **1.13 (1.12**–**1.13)** |
| Medication for cardiovascular disease | **1.19 (1.13**–**1.26)** | **1.19 (1.12**–**1.27)** | **2.21 (2.10**–**2.33)** | **1.67 (1.56**–**1.78)** | **1.14 (1.04**–**1.25)** | **1.28 (1.15**–**1.43)** | **7.85 (7.31**–**8.43)** | **1.65 (1.52**–**1.80)** |
| **BMI** |  |  |  |  |  |  |  |  |
| Underweight | 1.12 (0.82–1.51) | 1.03 (0.76–1.40) | 0.96 (0.69–1.35) | 1.00 (0.72–1.40) | 1.11 (0.70–1.77) | 1.08 (0.68–1.72) | **2.12 (1.66**–**2.71)** | **2.70 (1.94**–**3.77)** |
| Overweight | **1.23 (1.14**–**1.32)** | **1.28 (1.19**–**1.38)** | **1.37 (1.26**–**1.49)** | **1.21 (1.11**–**1.32)** | 1.03 (0.90–1.17) | 1.08 (0.94–1.23) | **1.11 (1.01**–**1.21)** | **0.82 (0.75**–**0.91)** |
| Obesity | **1.83 (1.70**–**1.97)** | **1.76 (1.64**–**1.92)** | **1.65 (1.52**–**1.79)** | **1.30 (1.19**–**1.42)** | 1.11 (0.97–1.27) | 1.11 (0.97–1.28) | **1.31 (1.20**–**1.43)** | **1.13 (1.02**–**1.25)** |
| Missing | **1.61 (1.50**–**1.74)** | **1.50 (1.38**–**1.64)** | 1.08 (1.00–1.17) | **1.13 (1.03**–**1.24)** | 0.94 (0.83–1.06) | 0.94 (0.82–1.08) | **0.86 (0.79**–**0.93)** | 1.08 (0.97–1.20) |
| **Smoking habits** |  |  |  |  |  |  |  |  |
| Former smoking | 1.02 (0.95–1.09) | 1.00 (0.94–1.08) | **1.28 (1.18**–**1.38)** | **1.12 (1.04**–**1.21)** | **0.87 (0.77**–**1.00)** | 0.90 (0.79–1.03) | **1.34 (1.23**–**1.45)** | 1.07 (0.97–1.17) |
| Current smoking | **1.51 (1.38**–**1.67)** | **1.45 (1.32**–**1.61)** | 0.91 (0.82–1.01) | 1.02 (0.91–1.14) | **0.58 (0.47**–**0.71)** | **0.56 (0.46**–**0.69)** | **0.85 (0.75**–**0.97)** | **2.53 (2.20**–**2.93)** |
| Missing | **1.32 (1.23**–**1.41)** | **1.20 (1.10**–**1.29)** | 0.96 (0.90–1.02) | 1.05 (0.97–1.14) | **0.86 (0.78**–**0.96)** | 0.91 (0.81–1.03) | **1.22 (1.14**–**1.30)** | **1.46 (1.33**–**1.60)** |

Abbreviation: ACC, asthma/COPD clinic; BMI, Body Mass Index. Notes: Presented as Odds Ratios with 95 % Confidence Intervals; Bold font symbolizes a p-value < 0.05.
